# Diagnostic potential of WNT signaling gene methylation in pulmonary tuberculosis

**DOI:** 10.3389/fimmu.2025.1639997

**Published:** 2025-11-18

**Authors:** Hua Wang, Chao-Cai Wang, Qian Huang, Yu Xiao, Yin Han, Lei Xie, Yan Liu, Yan-Jun Xiong

**Affiliations:** 1 Department of tuberculosis, Anhui Chest Hospital, Hefei, Anhui, China; 2 Department of Infection Disease, Qinghai Center for Disease Prevention and Control, Xining, China; 3 Department of Epidemiology and Biostatistics, School of Public Health, Anhui Medical University, Hefei, Anhui, China; 4 Department of Public Health, Medical College, Qinghai University, Xining, China

**Keywords:** pulmonary tuberculosis, DNA methylation, WNT signaling pathway genes, tuberculosis, epigenetics

## Abstract

**Objectives:**

The WNT signaling pathway plays important roles in the pathophysiology of pulmonary tuberculosis (PTB). This study aimed to assess the association of the DNA methylation levels of WNT signaling pathway genes and PTB susceptibility, and determine whether the methylation levels might serve as valuable biomarkers for PTB diagnosis.

**Methods:**

We collected blood samples from 98 PTB patients and 96 normal controls, and extracted DNA samples. The methylation levels of promoter region in WNT signaling pathway genes (*SFRP1*, *WNT3A*, *CTNNB1*, *WIF-1*, *DKK-1*, *LRP5*, *LRP6*) were detected by MethylTarget technique.

**Results:**

We found that the methylation levels of *SFRP1*, *WNT3A*, *CTNNB1*, *DKK-1*, *LRP6* genes were significantly decreased in the peripheral blood of PTB patients when compared to normal controls, while *WIF-1*, *LRP5* genes methylation levels showed no significant difference between PTB patients and controls. In PTB patients, the increased *SFRP1* methylation level was significantly correlated with drug-induced liver injury, pulmonary infection, and the decreased *WNT3A, CTNNB1* methylation levels were respectively significantly associated with drug resistance, fever. For diagnosing PTB, the *CTNNB1* methylation level demonstrated a relatively higher diagnostic value, achieving an AUC of 0.706. Subsequently, the AUC of *WNT3A*, *DKK-1*, *LRP6* was 0.660, 0.628, 0.621. The diagnostic value was slightly enhanced when *CTNNB1* combined with *WNT3A*, *DKK-1*, *LRP6*, with an AUC of 0.710.

**Conclusion:**

The methylation levels of *SFRP1*, *WNT3A*, *CTNNB1*, *DKK-1*, *LRP6* genes were significantly decreased in PTB patients, and single gene or multiple genes methylation panels might serve as potential diagnostic biomarkers for PTB.

## Introduction

Tuberculosis (TB) is a common and severe infectious disease caused by Mycobacterium tuberculosis (MTB), and it is regarded as a major global public health concern. Pulmonary tuberculosis (PTB) is the most common type. According to latest Global Tuberculosis Report, there are about 10.8 million new TB cases worldwide in 2023, and approximately 1.25 million people died from this disease (1). At present, only about 5-10% population infected with *MTB* would eventually develop active TB (2). Individual susceptibility varies might be related to multiple factors, such as nutritional status, immune function and genetic background (3, 4). Many genetic association studies had already identified some candidate genes related to TB susceptibility (5, 6). However, this only explained part of the genetic causes of TB, and the potential roles of other genetic mechanisms (epigenetic modifications, *etc.*) in TB needed to be further explored.

As the most common form of epigenetic modification, the dynamic regulation of DNA methylation modulated the immune response of *MTB*, thereby influencing the susceptibility and development of PTB (7, 8). Our previous study found higher methylation levels of *B7-H4* and *BTLA* genes in PTB patients (9). CpG sites in genome usually exhibited abnormal methylation modifications during early stages of disease, which had led to increasing emphasis on DNA methylation in the field of disease diagnosis. Moreover, DNA methylation biomarker had greater stability and could be detected in blood when compared with other biomarkers (10, 11). Hence, it was of great significance to search for methylation biomarkers that could be used for the early diagnosis of PTB.

Molecular pathways that regulate immune responses are a major focus of research in the study regarding PTB pathogenesis, including WNT pathways (12, 13). Recent evidences indicated that WNT signaling pathway was a crucial transduction cascade reaction that regulated the host’s immune response to microbial pathogens infection, and exerted significant pathogenic roles in the development of PTB (14). This study also found that the mRNA levels of several WNT related genes (*CTNNB1*, *SFRP1*, *WNT3A*) in patients with PTB were significantly elevated (14). *Wu et al.* demonstrated that activation of the Wnt/β−catenin signaling pathway induced apoptosis in macrophages infected with MTB and enhanced the production of TNF−α and IL−6 (15). In addition, genetic variations in WNT signaling pathway genes were also associated with susceptibility to PTB in many studies (14, 16).

Abnormal methylation levels of WNT signaling pathway genes had been found to be associated with the pathogenesis of various diseases. Our recent study found that multiple variants in WNT signaling pathway genes (*SFRP1*, *WNT3A*, *CTNNB1*, *WIF-1*, *DKK-1*, *LRP5*, *LRP6*) were linked to some clinical characteristics of PTB patients (16). Therefore, we hypothesized that the methylation levels of WNT signaling pathway genes might also be associated with the development of PTB. In this study, we detected and compared the WNT signaling pathway genes methylation levels in the peripheral blood of PTB patients and controls, and analyzed whether the abnormal methylation levels could serve as biomarkers for the diagnosis of PTB.

## Materials and methods

### Study participants

In this case-control study, the patients with PTB were selected from Anhui Chest Hospital between January 2023 and February 2024, while the age- and gender-matched healthy volunteers were included from the health examine center in the same area as control group. PTB patients were diagnosed based on the Diagnosis for PTB (WS 288-2017) and the Classification of TB (WS 196-2017) from the Health Industry Standard of the People’s Republic of China. For PTB patients, the diagnostic criteria mainly included: (1) positive pathogen diagnosis (bacterial culture, sputum smear examination, GeneXpert MTB/RIF test); (2) positive immunological diagnosis (interferon-γ release assay); (3) typical clinical manifestations and imaging examination. The exclusion criteria of PTB patients in this study comprised HIV positive, pneumonia, immune deficiency, and other infectious diseases. Controls group were required to had no TB-related symptoms, no history of infectious diseases, and a negative interferon-γ release assay result.

The design and implementation of this study were in line with the principles of the Helsinki Declaration and approved by the Ethics Committee of Anhui Chest Hospital. After obtaining informed consent, we collected demographic characteristics and peripheral blood samples from all study subjects, as well as the clinical data of PTB patients, including fever, drug resistance, drug-induced liver injury (DILI), pulmonary infection, total bilirubin (TBIL), erythrocyte sedimentation rate (ESR), aspartate aminotransferase (AST), alanine aminotransferase (ALT), *etc.*

### DNA extraction and bisulfite treatment

Approximately 3 mL of peripheral blood was collected from study subjects, and genomic DNA was extracted from peripheral blood lymphocytes using the Flexi Gene-DNA kit (Quona Company, Valencia, California) according to the manufacturer’s instructions. Then, the extracted DNA sample was quantified using a NanoDrop 2000 spectrophotometer. Finally, the DNA sample was subjected to bisulfite conversion using the EZ DNA Methylation Gold kit (Zymo Research, Orange, CA, USA) in accordance with the manufacturer’s instructions.

### Methylation detecting

The promoter regions of WNT related genes (*SFRP1*, *WNT3A*, *CTNNB1*, *WIF-1*, *DKK-1*, *LRP5*, *LRP6*) were subjected to target region methylation sequencing using MethylTarget, and this was carried out with the assistance of the Shanghai GeneCowin Biotechnology. The CpG islands in the promoter region of these genes were sequenced with the Illumina HiSeq (Illumina), and the primers were designed to amplify the specific target fragments from the bisulfite-converted DNA (**Supplementary Table S1**).

Two sequential polymerase chain reaction (PCR) amplifications were conducted, and the main instruments, reagents used in this experiment were listed in **Supplementary Table S2**. The initial amplification employed specific primers to enrich bisulfite-converted target fragments from DNA sample under optimized conditions. The cycle conditions of PCR reactions were set as follows: 95°C for 2 min, 11 cycles at 94°C for 20 sec and 63°C for 40 sec, 72°C for 1 min, 24 cycles at 94°C for 20 sec and 65°C for 30 sec and 72°C for 1 min, 72°C for 2 min. Subsequently, index PCR was conducted to pool all enriched products, amplifying them with indexed primers to append platform-compatible tag sequences (Illumina HiSeq compatible). The PCR products were size-separated using agarose gel electrophoresis and purified by TIANGENG Gel Extraction Kit, and subjected to high-throughput paired-end sequencing (2 × 150 bp) on the Illumina HiSeq platform to generate FastQ files. At this stage, the cycle conditions of PCR reactions were set as follows: 95°C for 30 sec, 11 cycles at 98°C for 10 sec and 65°C for 30 sec, and 72°C for 30 sec, 72°C for 5 min. The methylation level of certain specific fragment was obtained by calculating the mean methylation levels of all CpG sites on this fragment.

### Enzyme-linked immunosorbent assay

Plasma was isolated from peripheral blood by centrifugation at 3000 r.p.m. for 10 min and stored at -80°C until further analysis. The levels of SFRP1, WNT3A, CTNNB1, DKK−1, and LRP6 genes were measured using ELISA kits (Fine Biotech Co., Ltd., China) following the manufacturer’s instructions. The genes expression levels were expressed in nanograms per milliliter (ng/mL).

### Statistical analysis

The methylation levels were expressed as median value and interquartile range, and Mann-Whitney *U* test was used to compare the methylation levels between two different groups. The correlation between the methylation levels and laboratory data, expression levels was evaluated through Spearman’s rank correlation analysis. The diagnostic value of WNT signaling pathway gene methylation levels as biomarkers for PTB was evaluated through receiver operating characteristic (ROC) analysis and the area under the curve (AUC). The sensitivity, specificity, and cutoff values from ROC analysis was also calculated. All statistical analyses were conducted by SPSS statistics 26.0, and *P* value was less than 0.05 was considered statistically significant.

## Results

### Differences in WNT signaling pathway genes methylation levels between PTB and controls

This study enrolled a total of 194 subjects, comprising 98 PTB patients and 96 normal controls. The PTB patients had a mean age of 43.52 ± 17.59 years, with 45 females and 53 males. The control group exhibited a mean age of 43.44 ± 4.53 years, with 44 females and 52 males. We found that there was no statistically significant difference in the distribution of age and gender between these two groups.

As shown in **Supplementary Table S3**, this study first analyzed the methylation levels of multiple detected specific target fragments of WNT signaling pathway genes between PTB patients and controls. In comparison to controls, PTB patients had significant decrease in the methylation levels of *SFRP1_1*, *SFRP1_2*, *WNT3A_1*, *WNT3A_2*, *CTNNB1_1*, *CTNNB1_2*, *DKK-1_1*, *DKK-1_2*, *LRP6_2* (*P =* 0.015, *P =* 0.022, *P =* 0.002, *P* < 0.001, *P* < 0.001, *P* < 0.001, *P =* 0.018, *P =* 0.002, *P =* 0.003, respectively), and exhibited elevated methylation of *LRP6_1* (*P =* 0.002). No significant differences were observed in the *WIF-1_1*, *WIF-1_2*, *LRP5_1*, *LRP5_2*, *LRP6_3* methylation levels (*P* > 0.05).

**Figure 1:**
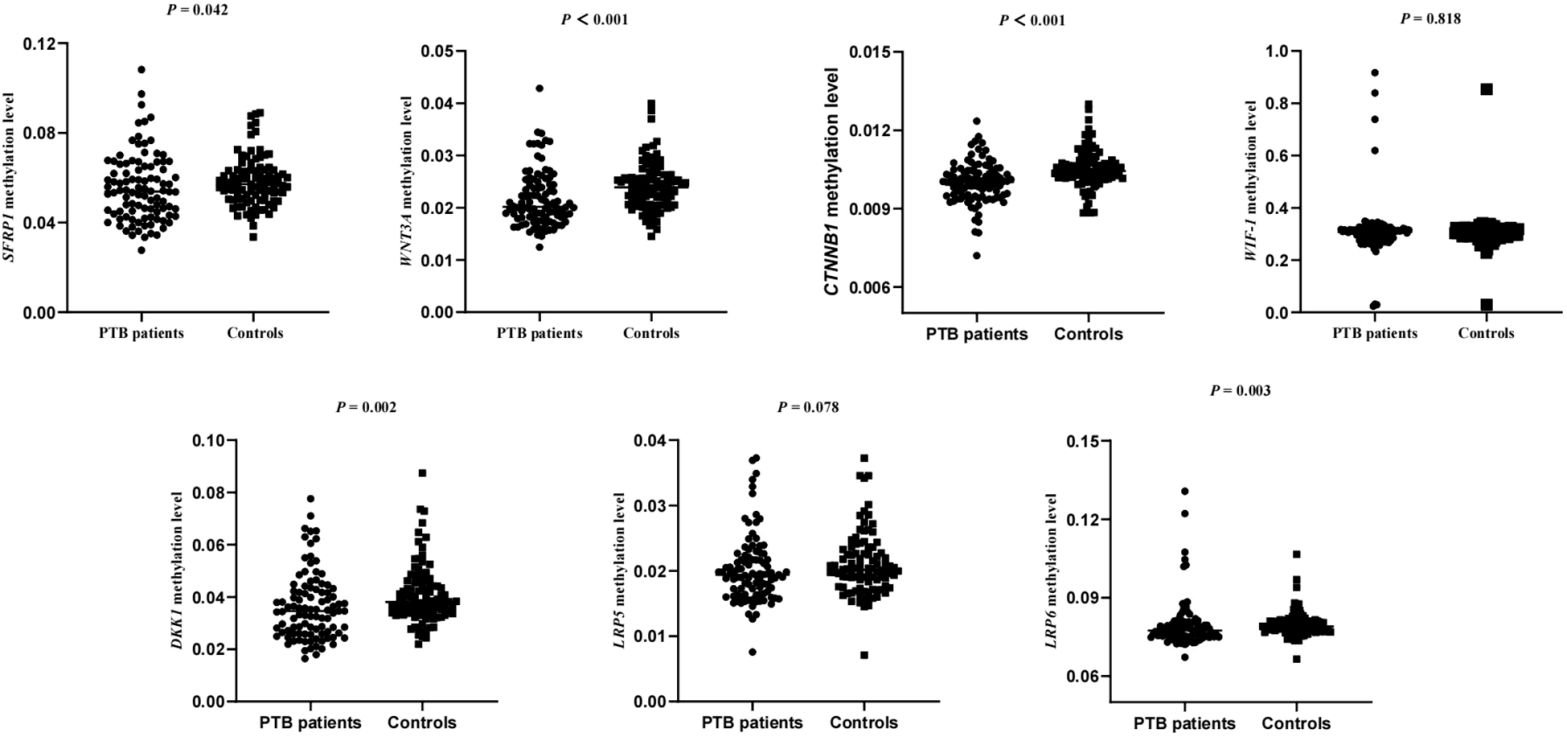
The methylation levels of *WNT* signaling pathway genes in PTB patients and normal controls.

Since each gene might contain more target fragments, the mean methylation level of all CpG sites on the detected specific fragments was calculated as the methylation level of each gene. Hence, we conducted further investigations to assess the association between the WNT signaling pathway genes methylation levels and the patients with PTB. The results were shown in **Figure 1**, and found that PTB patients exhibited decreased methylation levels of *SFRP1*, *WNT3A*, *CTNNB1*, *DKK-1*, *LRP6* (*P =* 0.042, *P* < 0.001, *P* < 0.001, *P =* 0.002, *P =* 0.003, respectively). In contrast, the *WIF-1*, *LRP5* methylation levels in PTB patients showed no statistically significant difference compared to that in normal controls.

The relationship between the methylation levels of *WNT* signaling pathway genes and gender in PTB patients was also analyzed. The results showed that the methylation levels of *WIF1* and *DKK-1* in male PTB patients were significantly lower than those in female patients, although no statistically significant differences were observed for the other genes (**Supplementary Table S4**).

### Correlation of WNT signaling pathway genes methylation levels with common clinical manifestations among PTB patients

Considering several common clinical features of PTB patients, such as fever, drug resistance, liver damage, and it was necessary to determine whether these genes methylation levels affect the occurrence of the clinical manifestations. Hence, we assessed the possible relationship between *SFRP1*, *WNT3A*, *CTNNB1*, *DKK-1*, *LRP6* methylation levels and several clinical manifestations among PTB patients. As illustrated in **Table 1**, the results suggested that PTB patients with DILI, pulmonary infection respectively exhibited significantly increased *SFRP1* methylation level than that in those patients without DILI, pulmonary infection (*P =* 0.001, *P =* 0.026). Moreover, the decreased *WNT3A, CTNNB1* methylation levels were respectively significantly associated with drug resistance, fever in PTB patients (*P* = 0.012, *P =* 0.011). However, the *DKK-1*, *LRP6* methylation levels were not related to above clinical features of PTB patients.

**Table 1:** Association between the methylation levels of WNT signaling pathway genes and clinical characteristics among PTB patients.

Group	N	*SFRP1* methylation level	*P* value	*WNT3A* methylation level	*P* value	*CTNNB1* methylation level	*P* value	*DKK-1* methylation level	*P* value	*LRP6* methylation level	*P* value
Fever			0.651		0.459		**0.011**		0.322		0.141
Yes	16	0.0479(0.0404, 0.0706)		0.0190(0.0166, 0.0280)		0.0097(0.0094, 0.0099)		0.0271(0.0230, 0.0474)		0.0753(0.0736, 0.0807)	
No	82	0.0541(0.0451, 0.0649)		0.0207(0.0181, 0.0246)		0.0101(0.0095, 0.0105)		0.0348(0.0276, 0.0424)		0.0775(0.0752, 0.0801)	
Drug resistance			0.626		**0.012**		0.029		0.114		0.093
Yes	12	0.0535(0.0415, 0.0613)		0.0180(0.0161, 0.0197)		0.0096(0.0094, 0.0100)		0.2810(0.2380, 0.3592)		0.0747(0.0725, 0.0807)	
No	86	0.0539(0.0449, 0.0661)		0.0209(0.0182, 0.0254)		0.0100(0.0095, 0.0105)		0.0351(0.0266, 0.0433)		0.0775(0.0752, 0.0807)	
DILI			**0.001**		0.125		0.095		0.106		0.085
Yes	13	0.0664(0.0570, 0.0802)		0.0237(0.0189, 0.0296)		0.0104(0.0097, 0.0109)		0.0400(0.0294, 0.0580)		0.0808(0.0750, 0.0854)	
No	85	0.0524(0.0424, 0.0619)		0.0200(0.0177, 0.0244)		0.0100(0.0094, 0.0104)		0.3426(0.2603, 0.4200)		0.0771(0.0749, 0.0796)	
Pulmonary infection			**0.026**		0.124		0.172		0.065		0.064
Yes	13	0.0654(0.0522, 0.0743)		0.0225(0.0191, 0.0287)		0.0095(0.0094, 0.0105)		0.0417(0.0343, 0.0476)		0.0803(0.0759, 0.0843)	
No	86	0.0534(0.0431, 0.0623)		0.0200(0.0175, 0.0244)		0.0100(0.0096, 0.0104)		0.0335(0.0259, 0.0419)		0.0767(0.0750, 0.0798)	
Sputum smear-positive			0.214		0.053		0.713		0.143		0.051
Yes	34	0.0529(0.0412, 0.0592)		0.0194(0.0171, 0.0232)		0.0100(0.0095, 0.0104)		0.0308(0.0241, 0.0425)		0.0759(0.0745, 0.0794)	
No	54	0.0542(0.0437, 0.0672)		0.0210(0.0184, 0.0262)		0.0100(0.0094, 0.0105)		0.0355(0.0279, 0.0447)		0.0780(0.0751, 0.0820)	

Bold value means *P* < 0.05.

N, number; DILI, drug-induced liver injury.

This study also evaluated the correlation between the methylation levels of WNT signaling pathway genes and several laboratory indicators of PTB patients, such as ESR, TBIL, ALT, and AST, while no correlation reached statistical significance (**Table 2**).

**Table 2:** The correlation between the methylation levels of WNT signaling pathway genes and ESR, TBIL, ALT, AST among PTB patients.

Clinical parameters	*SFRP1* methylation level		*WNT3A* methylation level		*CTNNB1* methylation level		*DKK-1* methylation level		*LRP6* methylation level	
	*r_s_*	*P* value	*r_s_*	*P* value	*r_s_*	*P* value	*r_s_*	*P* value	*r_s_*	*P* value
ESR	-0.515	0.616	-0.107	0327	0.014	0.900	-0.102	0.348	-0.184	0.091
TBIL	0.100	0.331	0.039	0.706	0.108	0.295	0.015	0.885	0.046	0.659
ALT	0.168	0.098	0.108	0.291	-0.077	0.451	0.078	0.444	0.147	0.147
AST	0.098	0.335	-0.009	0.933	0.121	0.237	0.043	0.671	-0.007	0.942

*r_s_*, Spearman’s rank correlation coefficient; TBIL, total bilirubin; ESR, erythrocyte sedimentation rate; AST, aspartate aminotransferase; ALT, alanine aminotransferase.

### Diagnostic value of WNT signaling pathway genes methylation level as biomarkers for PTB

**Figure 2:**
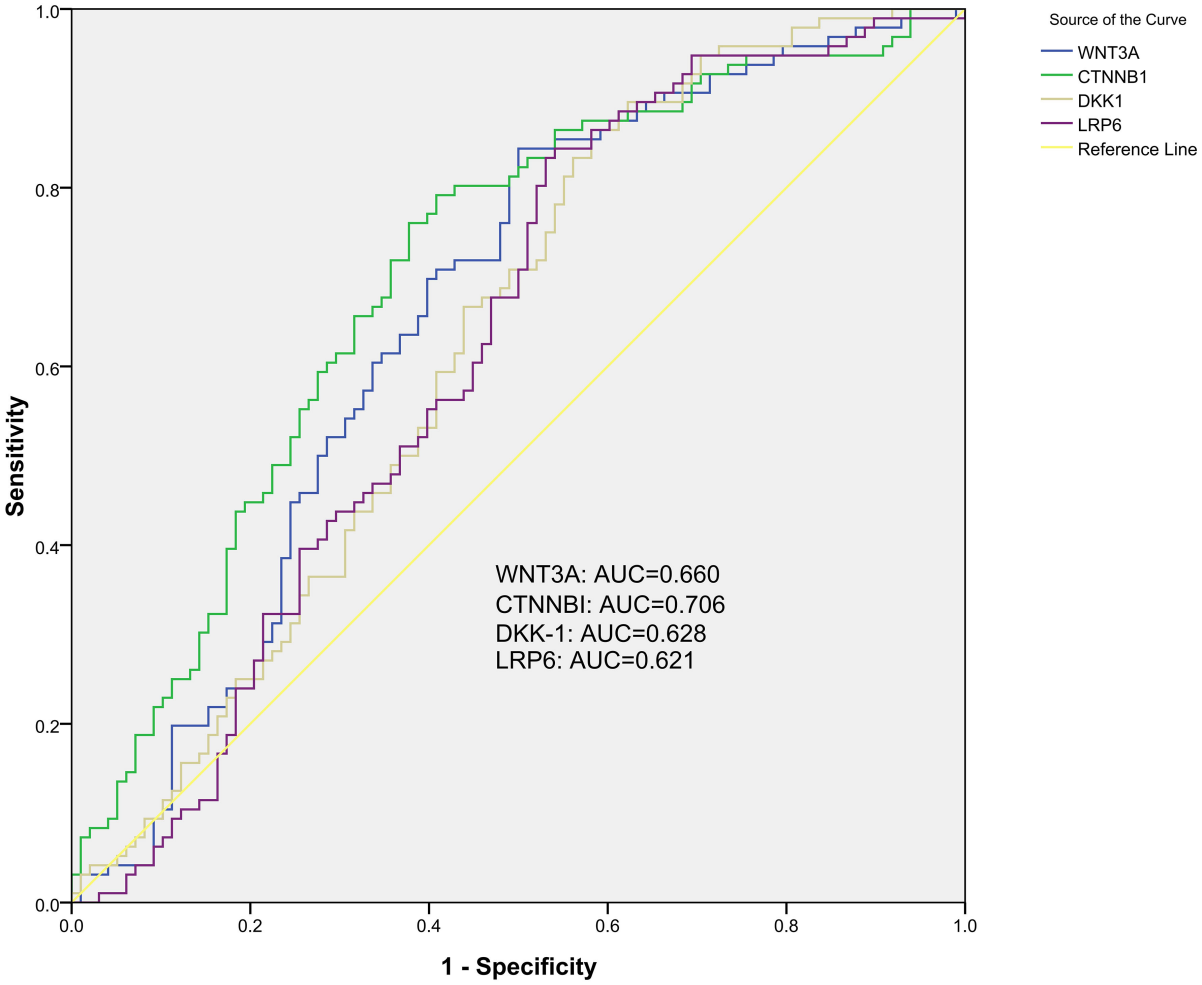
The diagnostic value of single gene methylation level in PTB with ROC curve and AUC.

To assessed the potential value of the five WNT signaling pathway genes as diagnostic biomarkers for PTB, the ROC curve analysis was performed. For discriminating PTB patients from normal controls, ROC curve analysis indicated that *WNT3A*, *CTNNB1*, *DKK-1*, *LRP6* methylation levels might be used as diagnostic biomarkers. The AUC of *CTNNB1*, *WNT3A*, *DKK-1*, *LRP6* was 0.706 (95% CI: 0.632, 0.780), 0.660 (95% CI: 0.582, 0.738), 0.628 (95% CI: 0.548, 0.707), 0.621 (95% CI: 0.541, 0.701), and *CTNNB1* methylation level seemed to had relatively higher diagnostic value (**Figure 2**). The sensitivity, specificity of above diagnostic biomarkers were shown in **Supplementary Table S5**.

**Figure 3:**
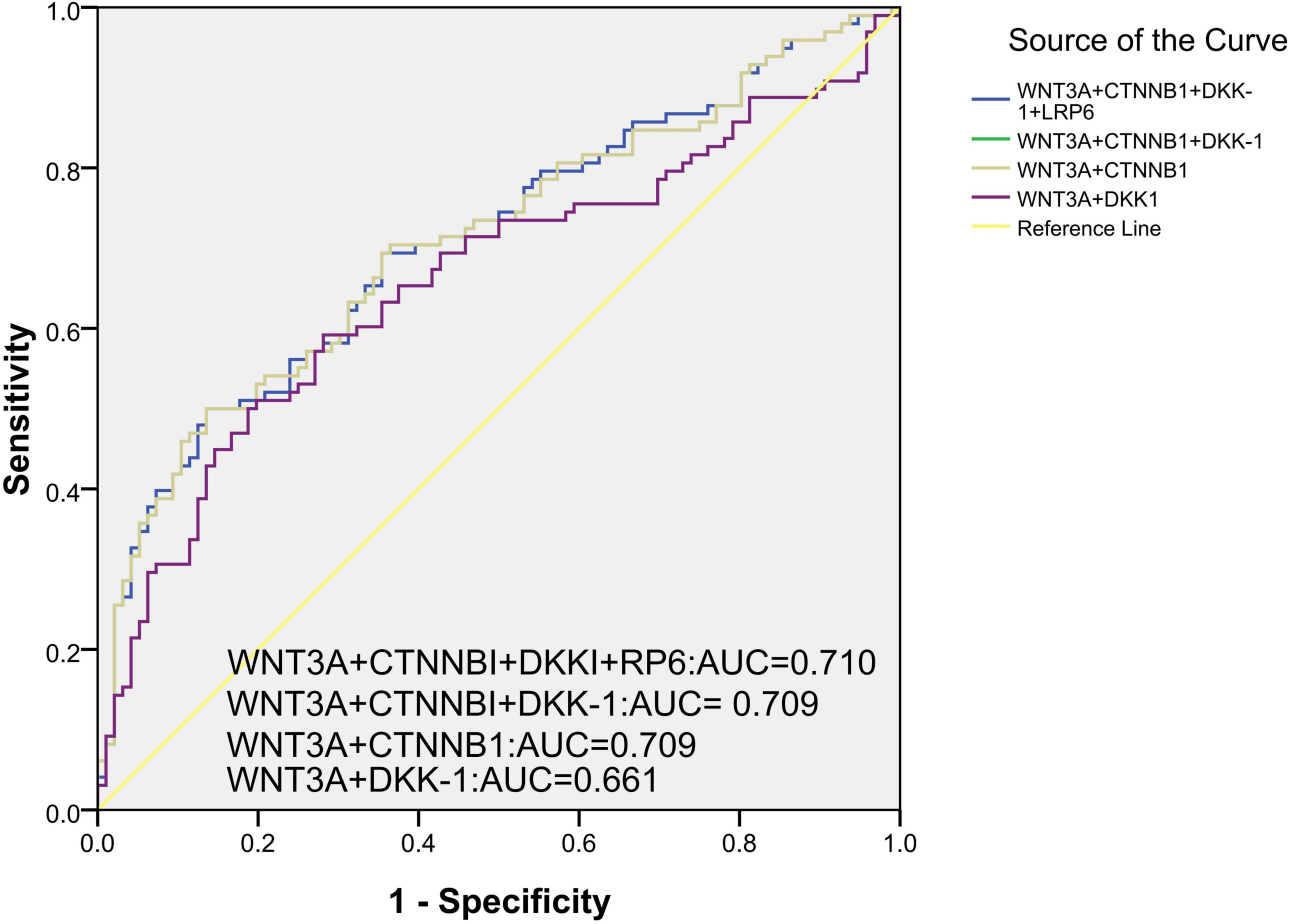
The combined diagnostic value of multiple gene methylation levels in PTB with ROC curve and AUC.

This study also further explored the comprehensive diagnostic value of these genes methylation levels for PTB, and identified three combined diagnostic models worthy of attention, including *WNT3A*+*CTNNB1*+*DKK-1*+*LRP6*, *WNT3A*+*CTNNB1*+*DKK-1, WNT3A*+*CTNNB1*. The AUC of these diagnostic models was 0.710 (95% CI: 0.637, 0.783), 0.709 (95% CI: 0.636, 0.782), 0.709 (95% CI: 0.636, 0.782) (**Figure 3**). The sensitivity, specificity of above diagnostic biomarkers were shown in **Supplementary Table S4**.

### Associations between DNA methylation haplotypes and PTB patients

For every specific target fragment of these genes, this study also identified multiple DNA methylation haplotypes, and further analyzed the relationship between the frequency of these methylation haplotypes and PTB patients. Compared with normal controls, the frequencies of five DNA methylation haplotypes (one methylation haplotypes in *CTNNB1_1*, *CTNNB1_2*, *DKK-1_1*, *DKK-1_2*, *LRP6_1*) were significantly increased in PTB patients, while the the frequencies of fifteen DNA methylation haplotypes (one methylation haplotypes in *DKK-1_1*, *LRP6_2*, *LRP6_3*, *WNT3A_2*, two methylation haplotypes in *CTNNB1_2*, *DKK-1_2*, *LRP6_1*, three methylation haplotypes in *CTNNB1_1*) were significantly lower. The abnormal DNA methylation haplotypes were summarized in **Supplementary Table S6**.

### Associations between WNT signaling pathway genes expression level and PTB patients

**Figure 4:**
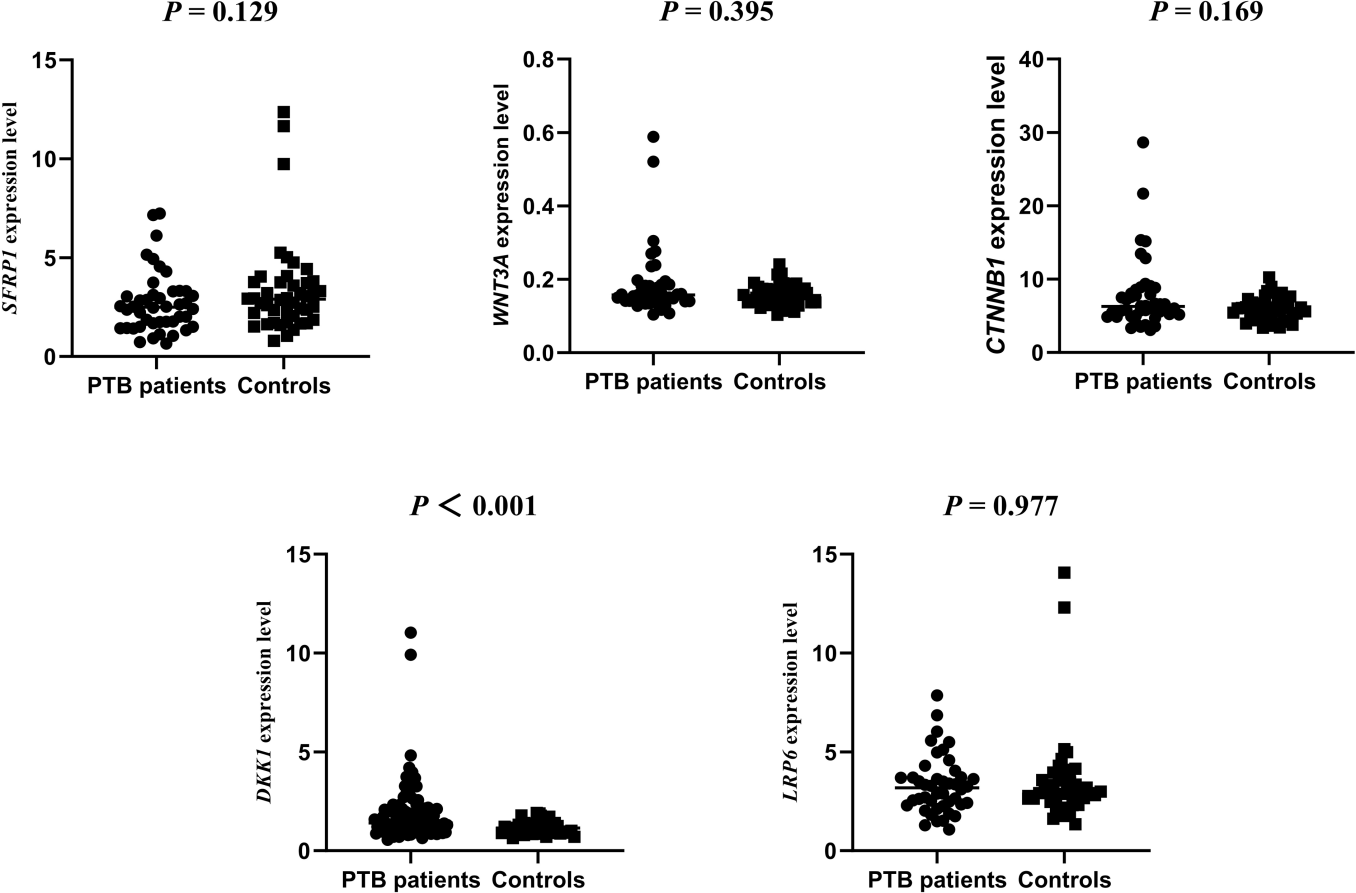
The expression levels of *SFRP1*, *WNT3A*, *CTNNB1*, *DKK-1*, *LRP6* genes in PTB patients and normal controls.

We assessed the *SFRP1*, *WNT3A*, *CTNNB1*, *DKK−1*, and *LRP6* genes expression levels in 44 PTB patients and 44 controls. The results showed that the expression level of *DKK−1* was significantly higher in PTB patients than in controls, while no other significant differences was found (*P* < 0.001) (**Figure 4**). The possible associations between *SFRP1, WNT3A, CTNNB1, DKK−1*, and *LRP6* genes methylation levels and their respective expression among PTB patients were also evaluated. The results found that the methylation levels of these five genes were not correlated with their respective expression levels in PTB patients (**Supplementary Table S7**).

## Discussion

In recent years, although the incidence of PTB has shown a downward trend and progress was made in PTB diagnosis, more than one-third of TB cases still fail to receive timely and effective identification. Epigenetic modifications represented a critical mechanism for regulating gene expression, and relevant studies had shown that DNA methylation not only played a significant role on the development of various diseases, including autoimmune diseases, infectious diseases, and cancers (17–19), but also could offer a new avenue for the early diagnosis of these diseases. For instance, the DNA methylation status of SHOX2/PTGER4 in plasma were proved to be valuable biomarkers for diagnosing lung cancer (20), and had been practically applied in clinical settings. In regard to infectious diseases, *Zhang* et al. identified several specific DNA methylation markers in *TSPAN4* gene from peripheral blood samples, holding promise for enhancing the diagnostic accuracy of PTB (21). Our latest research also indicated that the *ERAP2* methylation level was significantly higher in PTB patients when compared to controls, while the *ERAP1* methylation level was decreased (22). However, there was no research exploring the WNT related genes methylation in PTB. To our knowledge, this study was the first to detect the methylation levels of WNT signaling pathway genes in the peripheral blood of PTB patients. The *SFRP1*, *WNT3A*, *CTNNB1*, *DKK-1*, *LRP6* genes methylation levels were significantly decreased in PTB patients, suggesting that the methylation of WNT related genes might be related to the pathogenesis of PTB, as well as served as possible biomarkers for diagnosing PTB.

The WNT signaling pathway mediated immune regulatory function during pathogen infection and inflammation, and triggered pro-inflammatory or anti-inflammatory responses according to the cellular and cytokine environment (23, 24). Current evidences indicated that WNT signaling pathway played crucial roles in connecting innate and adaptive immunity to combat MTB infection, and its activation could limit the pro-inflammatory response after MTB infection (25). As a core component of the Wnt/β-catenin pathway, β-catenin exerted the key role in the CD4^+^T cells from PTB patients and mice (26). The BATF2 had also been found to regulate the number and function of macrophages by targeting TTC23 through the Wnt/β-catenin pathway during MTB infection (27). Therefore, the functions of WNT signaling pathway influenced the pathogenesis of PTB. Notably, genetic variations in multiple genes in *WNT* signaling pathway also affected the susceptibility and clinical manifestations of PTB. Our previous research revealed that *WIF-1* rs3782499, *DKK-1* GA haplotype were significantly related to PTB susceptibility, and *WNT3A* rs3121310, *CTNNB1* rs2293303, *LRP5* rs556442, *LRP6* rs10743980 polymorphisms were respectively associated with DILI, sputum smear-positive, fever (16). Furthermore, the methylation status of multiple genes related to WNT pathway was also involved in the development process of various diseases (28, 29). The study by *Zou* et al. suggested that *DKK‐1* hypermethylation might be associated with the pathological bone formation in ankylosing spondylitis (AS) (28). Here, we found the methylation levels of *WNT* related genes (*SFRP1*, *WNT3A*, *CTNNB1*, *DKK-1*, *LRP6*) were significantly reduced in PTB patients when compared to controls. During the onset and treatment process of PTB, patients usually exhibited some specific clinical features, such as drug resistance, liver damage, positive sputum smear, which significantly affected the treatment strategies. This study also stratified the patients based on their clinical features and further analyzed the relationship between *WNT* genes methylation levels and these clinical manifestations. Our results suggested that higher level of *SFRP1* was associated with DILI, pulmonary infection, and decreased levels of *WNT3A, CTNNB1* were respectively associated with drug resistance, fever among PTB patients. Above findings demonstrated that the abnormal methylation status of these genes might be involved in PTB process and providing new insights into the pathogenesis of PTB. Furthermore, the abnormal methylation levels of WNT related genes could also be related to the occurrence of certain clinical manifestations of patients with PTB, offering a valuable direction for reducing the occurrence of clinical manifestations.

Previous studies had suggested that WNT signaling pathway genes methylation levels could be considered as potential clinical biomarkers in human diseases (30, 31). *Boughanem* et al. established the promoter methylation level of *SFRP2* gene as a valuable prognostic candidate for colorectal cancer when evaluated in peripheral blood, while as an indicator for treatment in tumors (30). Moreover, the serum *DKK-1* methylation level could be regarded as a potential biomarker for evaluating the radiographic progression of AS (28). In this study, we searched for DNA methylation markers suitable for PTB diagnosis in WNT signaling pathway genes. Our results found that *CTNNB1* methylation level might serve as a valuable diagnostic indicator, with an AUC of 0.706. Then, we further constructed a combined diagnostic indicator incorporating the methylation levels of four genes (CTNNB1, WNT3A, DKK-1, LRP6), which yielded an AUC of 0.710. In another study, *Zhang et al.* validated several methylated positions in *TSPAN4* gene using pyrosequencing to serve as biomarkers for PTB diagnosis, achieving an AUC of 0.83 (21). In comparison, the methylation detection of WNT-related genes had certain potential for clinical diagnosis but could not yet support independent clinical application at the current AUC values. Moreover, methylation detection was relatively more cost-effective than pathogen detection and immunological detection. Therefore, further research with a larger sample size was needed to verify and improve the accuracy of methylation detection in PTB diagnosis. Generally, DNA methylation haplotype analysis also provided a new perspective for studying the direction of methylation changes (32). In this study, we finally identified 5 elevated and 15 decreased DNA methylation haplotypes in PTB patients, which were distributed across 8 specific target fragments, including *CTNNB1_1*, *CTNNB1_2*, *DKK-1_1*, *DKK-1_2*, *WNT3A_2, LRP6_1, LRP6_2*, *LRP6_3.* The changes in these methylation haplotypes discovered in this study might indicate inflammation-related alterations in PTB, highlighting the need for functional experiments to further investigate the roles of methylation changes in PTB.

Although our study presented some meaningful findings, it is also necessary to acknowledge several limitations. First, the sample size of this study might be relatively small, which affect the stability of our results. Second, this was a single-center cross-sectional study lacking an independent validation cohort. Third, we were unable to fully adjust for several important confounding factors, including drug exposure and underlying comorbidities. Therefore, future prospective, multi-center studies with larger sample sizes were needed to validate and extend these findings, thus facilitating the development of a more accurate early diagnostic model for PTB.

In conclusion, our study showed that the methylation levels of *SFRP1*, *WNT3A*, *CTNNB1*, *DKK-1*, *LRP6* genes in *WNT* signaling pathway were significantly decreased in PTB patients, and *SFRP1, WNT3A, CTNNB1* methylation levels were associated with some clinical characteristics, such as DILI, pulmonary infection, drug resistance, *etc.* Moreover, the abnormal methylation levels of WNT signaling pathway genes might be used as possible biomarkers for the diagnosis of PTB.

## Data Availability

The original contributions presented in the study are publicly available. The data presented in the study are deposited in the dbSNP (1063448).
