# Supplementary material for: Diagnostic potential of WNT signaling gene methylation in pulmonary tuberculosis
Source: Front Immunol. 2025 Nov 18;16:1639997. doi: 10.3389/fimmu.2025.1639997 (PMC12669203; doi:10.3389/fimmu.2025.1639997)
Supplement: Supplementary file 1 [file Table1.doc]

**Table S1** The primers of specific target fragments in *WNT* signaling pathway genes

| Gene | Fragment | Forward primer | Reverse primer |
| --- | --- | --- | --- |
| SFRP1 | SFRP1_1 | GAGGTAGTTTTATTTTGGGGTTTG | ACCCCTACTCAACAAAAACTACCA |
|  | SFRP1_2 | GGAGGTTGTAGGGTTGGAGTG | CTTCTTTTTCTCCCCTTATCTCTTTC |
| WNT3A | WNT3A_1 | GYGTTAGTTTTTAGGGTTYGGTTTTT | AAAAACTCACTCACCACCAAATC |
|  | WNT3A_2 | GATTTGGTGGTGAGTGAGTTTT | CRACCTAAACAAATCCAAAATAACC |
| CTNNB1 | CTNNB1_1 | GTTTTGGGAGGGYGGAAGT | ACRACCCAAATCCAACAAAAA |
|  | CTNNB1_2 | GAGGGGGYGGTGGAAAGTAG | ACCTTCCCCAACCCAAAC |
| WIF1 | WIF1_1 | GGGGTAAATAGAGYGAGAATAGAAGAG | CCCAAAAATCTCTAAATACCCTTCT |
|  | WIF1_2 | AAATGTTGGGTGTYGGGTAAG | CCCTTTCAACCAATAAAATTCCTCT |
| DKK-1 | DKK-1_1 | TTTTTGTTGGGAGTGAGYGTTATTT | TCAAAATCAAAACATCCTCTAAATACC |
|  | DKK-1_2 | AYGAGGAGTGYGGTATTGATGAG | AAAAACTTTCAAAACTCACCATTTTTAC |
| LRP5 | LRP5_1 | AAATTGTTTYGTTGTGTGTTTTTG | TCCCCRCCCTACCCTAAAA |
|  | LRP5_2 | TTTATAGAGGTTTTATAGGTGATATATTTGGTTTY | ACCAAACRACCCCAACRAC |
| LRP6 | LRP6_1 | AGGAGTATATAGAAGTTGTAGGTTAGGA | ACCCCTTTCTTTCTTCTCTCRC |
|  | LRP6_2 | ATTTTGGGGGTAGTTTGTATTTTTG | CCCCAACCACCTAAAACTACC |
|  | LRP6_3 | GGTTGTAATTTTAGTATTTTGGGAGGT | CTCCTACTTCAACCTCCTAAATAACTAAAA |

**Table S2 The Basic Information of Instruments and Reagents**

| Instruments/Reagents names | Supplier |
| --- | --- |
| ABI 2720 Thermal Cycler | Applied Biosystems, Waltham, MA, USA |
| Eppendorf 5810R Centrifuge | Eppendorf, Hamburg, Germany |
| XiangYi H1650-W | XiangYi, Hunan, China |
| EP600 Gel electrophoresis | Shanghai Yupu Industrial, China |
| NanoDrop 2000 | NanoDrop technologies, Wilmington, DE, USA |
| Invitrogen Qubit 3.0 Spectrophotometer | Invitrogen, Carlsbad, CA, USA |
| Agilent 2100 bioanalyzer | Agilent Technologies, USA |
| Illumina Hiseq/Nova seq | Illumina, CA, USA |
| EZ DNA Methylation-Gold Kit | ZYMO, CA, USA |
| TIANGEN Gel Extraction kit | TIANGEN, Beijing，China |
| 10× Reaction buffer | TaKaRa, Dalian, China |
| HotStart Taq polymerase | TaKaRa, Dalian, China |
| Herculase® II Fusion DNA Polymerases | Agilent Technologies, CA, USA |

**Table S3** The methylation levels of specific target fragments in WNT signaling pathway genes among PTB patients and controls

Bold value means *P* < 0.05.

| Specific target fragments | PTB patients (n=98) | Controls (n=96) | P value |
| --- | --- | --- | --- |
| SFRP1_1 | 0.081(0.068, 0.097) | 0.087(0.079, 0.096) | **0.015** |
| SFRP1_2 | 0.026(0.020, 0.031) | 0.027(0.024, 0.032) | **0.022** |
| WNT3A_1 | 0.015(0.013, 0.018) | 0.017(0.015, 0.019) | **0.002** |
| WNT3A_2 | 0.024(0.021, 0.029) | 0.028(0.024, 0.031) | **< 0.001** |
| CTNNB1_1 | 0.009(0.009, 0.010) | 0.010(0.009, 0.010) | **< 0.001** |
| CTNNB1_2 | 0.011(0.010, 0.013) | 0.011(0.011, 0.012) | **< 0.001** |
| WIF1_1 | 0.025(0.021, 0.029) | 0.026(0.022, 0.029) | 0.306 |
| WIF1_2 | 0.788(0.727, 0.815) | 0.769(0.741, 0.828) | 0.951 |
| DKK-1_1 | 0.029(0.023, 0.035) | 0.031(0.028, 0.036) | **0.018** |
| DKK-1_2 | 0.040(0.029, 0.053) | 0.046(0.039, 0.056) | **0.001** |
| LRP5_1 | 0.019(0.016, 0.022) | 0.020(0.016, 0.024) | 0.061 |
| LRP5_2 | 0.020(0.017, 0.023) | 0.020(0.018, 0.023) | 0.379 |
| LRP6_1 | 0.011(0.009, 0.012) | 0.010(0.011, 0.013) | **0.002** |
| LRP6_2 | 0.023(0.018, 0.029) | 0.026(0.022, 0.031) | **0.003** |
| LRP6_3 | 0.243(0.230, 0.246) | 0.244(0.241, 0.248) | 0.182 |

**Table S4** The methylation levels of WNT signaling pathway genes in patients with PTB of different genders

| Gene | Male(n=53) | Female (n=42) | P value |
| --- | --- | --- | --- |
| SFRP1 | 0.054(0.045, 0.064) | 0.054(0.042, 0.067) | 0.895 |
| WNT3A | 0.020(0.017, 0.024) | 0.021(0.018, 0.026) | 0.241 |
| CTNNB1 | 0.010(0.009, 0.011) | 0.010(0.009, 0.011) | 0.581 |
| WIF1 | 0.300(0.275, 0.316) | 0.311(0.293, 0.323) | **0.043** |
| DKK-1 | 0.033(0.025, 0.040) | 0.038(0.028, 0.046) | **0.021** |
| LRP5 | 0.019(0.016, 0.021) | 0.020(0.017, 0.023) | 0.066 |
| LRP6 | 0.076(0.075, 0.080) | 0.078(0.075, 0.081) | 0.162 |

**Table S5** The sensitivity, specificity of each diagnostic biomarkers

| Indicators | AUC(95% CI:) | Cutoff value | Sensitivity | Specificity |
| --- | --- | --- | --- | --- |
| *CTNNB1* | 0.706 (0.632, 0.780) | 0.010 | 79.2% | 59.2% |
| *WNT3A* | 0.660 (0.582, 0.738) | 0.020 | 84.4% | 50.0% |
| *DKK-1* | 0.628 (0.548, 0.707) | 0.032 | 86.5% | 71.7% |
| *LRP6* | 0.621 (0.541, 0.701) | 0.076 | 83.3% | 46.9% |
| *WNT3A*+*CTNNB1*+*DKK-1*+*LRP6* | 0.710 (0.637, 0.783) | 0.591 | 50.0% | 86.5% |
| *WNT3A*+*CTNNB1*+*DKK-1* | 0.709 (0.636, 0.782) | 0.592 | 50.0% | 86.5% |
| *WNT3A*+*CTNNB1* | 0.709 (0.636, 0.782) | 0.592 | 50.0% | 86.5% |

**Table S6** Association between The DNA methylation haplotypes of WNT signaling pathway genes and PTB patients

| Gene fragment | Haplotype | PTB patients | Controls | *P* value |
| --- | --- | --- | --- | --- |
| CTNNB1_1 | TTTTTTTTTTTTTTTTTTT | 0.8429 | 0.8345 | **0.033** |
| CTNNB1_1 | CTTTTTTTTTTTTTTTTTT | 0.0118 | 0.0131 | 0.080 |
| CTNNB1_1 | TTTCTTTTTTTTTTTTTTT | 0.0124 | 0.0123 | 0.971 |
| CTNNB1_1 | TTTTTTTTTTTTTTTCTTT | 0.0105 | 0.0110 | 0.405 |
| CTNNB1_1 | TTTTTTTTTTTTTTTTTTC | 0.0101 | 0.0107 | 0.446 |
| CTNNB1_1 | TTTTTTTTTTTTTCTTTTT | 0.0097 | 0.0098 | 0.900 |
| CTNNB1_1 | TTTTTTTTTTTCTTTTTTT | 0.0072 | 0.0078 | 0.193 |
| CTNNB1_1 | TTTTTTTTTTTTTTTTTCT | 0.0066 | 0.0082 | **0.040** |
| CTNNB1_1 | TCTTTTTTTTTTTTTTTTT | 0.0071 | 0.0072 | 0.872 |
| CTNNB1_1 | TTTTTTTTCTTTTTTTTTT | 0.0068 | 0.0072 | 0.406 |
| CTNNB1_1 | TTTTTCTTTTTTTTTTTTT | 0.0067 | 0.0067 | 0.980 |
| CTNNB1_1 | TTTTTTCTTTTTTTTTTTT | 0.0059 | 0.0064 | 0.240 |
| CTNNB1_1 | TTCTTTTTTTTTTTTTTTT | 0.0065 | 0.0063 | 0.757 |
| CTNNB1_1 | TTTTTTTTTTTTTTTTCTT | 0.0063 | 0.0069 | 0.428 |
| CTNNB1_1 | TTTTTTTTTCTTTTTTTTT | 0.0059 | 0.0070 | **0.049** |
| CTNNB1_1 | TTTTTTTTTTTTCTTTTTT | 0.0064 | 0.0060 | 0.274 |
| CTNNB1_1 | TTTTTTTTTTCTTTTTTTT | 0.0061 | 0.0059 | 0.455 |
| CTNNB1_1 | TTTTTTTTTTTTTTCTTTT | 0.0061 | 0.0059 | 0.662 |
| CTNNB1_1 | TTTTTTTCTTTTTTTTTTT | 0.0059 | 0.0058 | 0.725 |
| CTNNB1_1 | TTTTCTTTTTTTTTTTTTT | 0.0048 | 0.0058 | **0.040** |
| CTNNB1_2 | TTTTTTTTTTTTTTTTTTTTT | 0.8056 | 0.7950 | **0.035** |
| CTNNB1_2 | TTTTTTCTTTTTTTTTTTTTT | 0.0506 | 0.0513 | 0.670 |
| CTNNB1_2 | TTTTTCTTTTTTTTTTTTTTT | 0.0128 | 0.0132 | 0.508 |
| CTNNB1_2 | TTTTTTTTTTTTTTTTTCTTT | 0.0074 | 0.0080 | 0.117 |
| CTNNB1_2 | TTTTTTTTTTTTCTTTTTTTT | 0.0068 | 0.0084 | **0.002** |
| CTNNB1_2 | TTTTTTTTTTTTTTTTTTTCT | 0.0069 | 0.0075 | 0.129 |
| CTNNB1_2 | TTCTTTTTTTTTTTTTTTTTT | 0.0072 | 0.0073 | 0.831 |
| CTNNB1_2 | TTTTTTTTTTTTTTTCTTTTT | 0.0071 | 0.0072 | 0.745 |
| CTNNB1_2 | TTTTTTTTTTTTTTTTTTCTT | 0.0069 | 0.007 | 0.844 |
| CTNNB1_2 | TTTCTTTTTTTTTTTTTTTTT | 0.0062 | 0.0069 | 0.054 |
| CTNNB1_2 | CTTTTTTTTTTTTTTTTTTTT | 0.0085 | 0.0072 | 0.559 |
| CTNNB1_2 | TTTTTTTTTTTTTCTTTTTTT | 0.0056 | 0.006 | 0.247 |
| CTNNB1_2 | TTTTTTTTTTTTTTTTCTTTT | 0.0056 | 0.0068 | **0.006** |
| CTNNB1_2 | TTTTTTTTTCTTTTTTTTTTT | 0.0057 | 0.0062 | 0.279 |
| CTNNB1_2 | TCTTTTTTTTTTTTTTTTTTT | 0.0056 | 0.0053 | 0.315 |
| CTNNB1_2 | TTTTCTTTTTTTTTTTTTTTT | 0.0053 | 0.0056 | 0.541 |
| CTNNB1_2 | TTTTTTTTTTTTTTTTTTTTC | 0.0051 | 0.0054 | 0.323 |
| CTNNB1_2 | TTTTTTTTTTTTTTCTTTTTT | 0.0050 | 0.0054 | 0.265 |
| DKK-1_1 | TTTTTTTTTTTTT | 0.7466 | 0.7404 | 0.380 |
| DKK-1_1 | TTTTTTTTTTTTC | 0.0525 | 0.0499 | 0.538 |
| DKK-1_1 | TTTTTTTTTTTCT | 0.0265 | 0.0234 | **0.023** |
| DKK-1_1 | TTTTTTCTTTTTT | 0.0178 | 0.0194 | 0.060 |
| DKK-1_1 | CTTTTTTTTTTTT | 0.0143 | 0.0155 | 0.089 |
| DKK-1_1 | TTTTTTTCTTTTT | 0.0112 | 0.0115 | 0.569 |
| DKK-1_1 | TTTTTTTTTTCTT | 0.0102 | 0.011 | 0.198 |
| DKK-1_1 | TTTTTTTTTCTTT | 0.0097 | 0.0083 | 0.269 |
| DKK-1_1 | TTCTTTTTTTTTT | 0.0067 | 0.0079 | **0.023** |
| DKK-1_1 | TTTTTTCTTTTCT | 0.0017 | 0.002 | 0.241 |
| DKK-1_1 | CTTTTTTTTTTCT | 0.0008 | 0.0015 | 0.286 |
| DKK-1_2 | TTTTTTTTTTTT | 0.7585 | 0.7326 | **0.017** |
| DKK-1_2 | CTTTTTTTTTTT | 0.018 | 0.0189 | 0.317 |
| DKK-1_2 | TTTTTTTCTTTT | 0.0179 | 0.0177 | 0.862 |
| DKK-1_2 | TTTTTCTTTTTT | 0.0146 | 0.0152 | 0.500 |
| DKK-1_2 | TTTTTTTTTCTT | 0.0127 | 0.0144 | **0.030** |
| DKK-1_2 | TTTTTTCTTTTT | 0.0119 | 0.0125 | 0.434 |
| DKK-1_2 | TTTCTTTTTTTT | 0.0122 | 0.0121 | 0.907 |
| DKK-1_2 | TCTTTTTTTTTT | 0.0107 | 0.0107 | 0.954 |
| DKK-1_2 | TTCTTTTTTTTT | 0.0100 | 0.0108 | 0.190 |
| DKK-1_2 | TTTTTTTTTTCT | 0.0140 | 0.0189 | 0.601 |
| DKK-1_2 | TTTTCTTTTTTT | 0.0094 | 0.0101 | 0.232 |
| DKK-1_2 | TTTTTTTTCTTT | 0.0069 | 0.0080 | **0.047** |
| DKK-1_2 | TTTTTTTTTTTC | 0.0071 | 0.007 | 0.813 |
| DKK-1_2 | TTCCCCTTTTTT | 0.0003 | 0.001 | 0.292 |
| LRP6_1 | TTTTTTTTTTTT | 0.8928 | 0.8860 | **0.049** |
| LRP6_1 | TTTTTTCTTTTT | 0.0133 | 0.0155 | **0.008** |
| LRP6_1 | TTTCTTTTTTTT | 0.0139 | 0.0122 | 0.442 |
| LRP6_1 | TTTTTTTTCTTT | 0.0106 | 0.0115 | 0.100 |
| LRP6_1 | TTTTTTTTTCTT | 0.0102 | 0.0111 | 0.156 |
| LRP6_1 | TTTTTTTCTTTT | 0.0082 | 0.0088 | 0.275 |
| LRP6_1 | TTTTTTTTTTCT | 0.0065 | 0.0074 | 0.063 |
| LRP6_1 | TTTTTCTTTTTT | 0.0067 | 0.0073 | 0.177 |
| LRP6_1 | TTTTCTTTTTTT | 0.006 | 0.0064 | 0.350 |
| LRP6_1 | TTTTTTTTTTTC | 0.0061 | 0.0059 | 0.861 |
| LRP6_1 | CTTTTTTTTTTT | 0.0054 | 0.0059 | 0.159 |
| LRP6_1 | TCTTTTTTTTTT | 0.0043 | 0.004 | 0.453 |
| LRP6_1 | TTCTTTTTTTTT | 0.0033 | 0.0045 | **< 0.001** |
| LRP6_1 | TTTCTTTCTTTT | 0.0002 | 0.0002 | 0.553 |
| LRP6_2 | TTTTTTTTTTTTTTTTTTT | 0.7125 | 0.7015 | 0.248 |
| LRP6_2 | CTTTTTTTTTTTTTTTTTT | 0.055 | 0.057 | 0.592 |
| LRP6_2 | TTTTTTCTTTTTTTTTTTT | 0.0277 | 0.026 | 0.356 |
| LRP6_2 | TCTTTTTTTTTTTTTTTTT | 0.0103 | 0.0117 | 0.284 |
| LRP6_2 | TTTTCTTTTTTTTTTTTTT | 0.01 | 0.0094 | 0.622 |
| LRP6_2 | TTTTTTTTTTTTTTTTTCT | 0.0123 | 0.0087 | 0.322 |
| LRP6_2 | TTTCTTTTTTTTTTTTTTT | 0.0077 | 0.0088 | 0.355 |
| LRP6_2 | TTTTTCTTTTTTTTTTTTT | 0.0071 | 0.009 | 0.064 |
| LRP6_2 | TTTTTTTCTTTTTTTTTTT | 0.0103 | 0.0073 | 0.292 |
| LRP6_2 | TTTTTTTTTTTTCTTTTTT | 0.0066 | 0.0079 | 0.136 |
| LRP6_2 | TTTTTTTTTTTTTCTTTTT | 0.0061 | 0.0063 | 0.786 |
| LRP6_2 | TTCTTTTTTTTTTTTTTTT | 0.0058 | 0.0073 | **0.024** |
| LRP6_2 | TTTTTTTTTTTTTTCTTTT | 0.0064 | 0.0064 | 0.964 |
| LRP6_2 | TTTTTTTTCTTTTTTTTTT | 0.0076 | 0.0062 | 0.378 |
| LRP6_2 | TTTTTTTTTTTTTTTCTTT | 0.0058 | 0.0057 | 0.862 |
| LRP6_2 | TTTTTTTTTTTTTTTTCTT | 0.0055 | 0.005 | 0.486 |
| LRP6_2 | CTTTTTCTTTTTTTTTTTT | 0.0045 | 0.0047 | 0.759 |
| LRP6_2 | TTTTTTTTTTTCTTTTTTT | 0.0036 | 0.0041 | 0.343 |
| LRP6_2 | CTTTTTTTTTTTTTTTTCT | 0.0007 | 0.0019 | 0.216 |
| LRP6_2 | CTTTTTTTTTTTTTCTTTT | 0.0004 | 0.0012 | 0.184 |
| LRP6_2 | TTTTTTTTCTTTTCTTTTT | 0.001 | 0.0001 | 0.361 |
| LRP6_3 | TTTTTTTTTTT | 0.0978 | 0.0969 | 0.473 |
| LRP6_3 | TTTCTTTTTTT | 0.0361 | 0.0364 | 0.661 |
| LRP6_3 | CTTTTTTTTTT | 0.031 | 0.0301 | 0.129 |
| LRP6_3 | TTTTTCTTTTT | 0.0263 | 0.0274 | **0.036** |
| LRP6_3 | TTTTTTTCTTT | 0.0233 | 0.0235 | 0.684 |
| LRP6_3 | TTTTTTTTTTC | 0.0164 | 0.0166 | 0.607 |
| LRP6_3 | TTTTTTCTTTT | 0.0161 | 0.0159 | 0.673 |
| SFRP1_1 | TTTTTTTTTTTTTTTTTTTTTTTT | 0.325 | 0.3342 | 0.434 |
| SFRP1_1 | CTTTTTTTTTTTTTTTTTTTTTTT | 0.0261 | 0.0248 | 0.212 |
| SFRP1_1 | TTTTTTTTTTTTTTTTTTTTTCTT | 0.019 | 0.0206 | 0.153 |
| SFRP1_1 | TTTTTTTCTTTTTTTTTTTTTTTT | 0.019 | 0.0173 | 0.134 |
| SFRP1_1 | TTTTTTTTTTTTTTTTTTTTCTTT | 0.0161 | 0.0162 | 0.928 |
| SFRP1_1 | TTTTTTTTTTTTTTTTTCTTTTTT | 0.0211 | 0.0134 | 0.231 |
| SFRP1_1 | TCTTTTTTTTTTTTTTTTTTTTTT | 0.0141 | 0.013 | 0.195 |
| SFRP1_1 | TTTTTTTTCTTTTTTTTTTTTTTT | 0.0116 | 0.0117 | 0.899 |
| SFRP1_1 | TTTTTTTTTTTTCTTTTTTTTTTT | 0.0107 | 0.0104 | 0.676 |
| SFRP1_1 | TTTTTTTTTTTTTTTTTTTTTTCT | 0.0119 | 0.0106 | 0.397 |
| SFRP1_1 | TTTTTTTTTTTTTTTCTTTTTTTT | 0.0112 | 0.0104 | 0.274 |
| SFRP1_1 | TTTTTTTTTTTCTTTTTTTTTTTT | 0.0076 | 0.0086 | 0.051 |
| SFRP1_1 | TTTCTTTTTTTTTTTTTTTTTTTT | 0.0078 | 0.0074 | 0.553 |
| SFRP1_1 | TTTTTTTTTTTTTTTTTTTTCCTT | 0.0074 | 0.0083 | 0.501 |
| SFRP1_1 | TTTTTTTTTTCTTTTTTTTTTTTT | 0.0113 | 0.0045 | 0.291 |
| SFRP1_1 | TTTTTTTTTTTTTTTTTTTCCTTT | 0.0045 | 0.0038 | 0.415 |
| SFRP1_1 | TTTTTTTTTTTTTTTTCTTTTTTT | 0.0033 | 0.0049 | 0.165 |
| SFRP1_1 | TTTTTTTTTTTTTTTTTTTCCCTT | 0.0035 | 0.0034 | 0.823 |
| SFRP1_1 | TTTTTTTTTTTTTTTCTCTTTTTT | 0.0021 | 0.002 | 0.832 |
| SFRP1_1 | CTTTTTTTTCTTCTTTTTTTTTTT | 0.0011 | 0.0001 | 0.340 |
| SFRP1_2 | TTTTTTTTTTTTTTTTTTTTTTTTT | 0.6924 | 0.6824 | 0.291 |
| SFRP1_2 | TTTTTTTTTTTTTTTCTTTTTTTTT | 0.0147 | 0.0179 | 0.056 |
| SFRP1_2 | TTTTTTTTTTTTTTTTTTTTTTCTT | 0.0163 | 0.0139 | 0.252 |
| SFRP1_2 | TTTTTTTTTTTTTTCTTTTTTTTTT | 0.0158 | 0.0134 | 0.105 |
| SFRP1_2 | TTTTTTTTTTTTTTTTTTCTTTTTT | 0.0115 | 0.0105 | 0.344 |
| SFRP1_2 | CTTTTTTTTTTTTTTTTTTTTTTTT | 0.0089 | 0.0099 | 0.217 |
| SFRP1_2 | TTTTTTTTTTTCTTTTTTTTTTTTT | 0.0114 | 0.0102 | 0.584 |
| SFRP1_2 | TTTTTTTTTTTTTTTTTTTTTCTTT | 0.009 | 0.0095 | 0.569 |
| SFRP1_2 | TTCTTTTTTTTTTTTTTTTTTTTTT | 0.008 | 0.0089 | 0.373 |
| SFRP1_2 | TTTTTTTTTTTTTTTTTTTTCTTTT | 0.0065 | 0.01 | 0.118 |
| SFRP1_2 | TTTTTTCTTTTTTTTTTTTTTTTTT | 0.0064 | 0.0108 | 0.052 |
| SFRP1_2 | TTTTTTTCTTTTTTTTTTTTTTTTT | 0.0064 | 0.0069 | 0.438 |
| SFRP1_2 | TTTTTTTTCTTTTTTTTTTTTTTTT | 0.0057 | 0.007 | 0.086 |
| SFRP1_2 | TTTTTTTTTTTTTTTTTTTCTTTTT | 0.006 | 0.0073 | 0.185 |
| SFRP1_2 | TTTTTTTTTCTTTTTTTTTTTTTTT | 0.0099 | 0.0062 | 0.272 |
| SFRP1_2 | TTTTTTTTTTTTCTTTTTTTTTTTT | 0.0055 | 0.0052 | 0.671 |
| SFRP1_2 | TTTTTCTTTTTTTTTTTTTTTTTTT | 0.0061 | 0.0052 | 0.279 |
| SFRP1_2 | TTTTTTTTTTTTTTTTCTTTTTTTT | 0.0049 | 0.0058 | 0.313 |
| SFRP1_2 | TTTCTTTTTTTTTTTTTTTTTTTTT | 0.0051 | 0.0053 | 0.717 |
| SFRP1_2 | TTTTCTTTTTTTTTTTTTTTTTTTT | 0.0045 | 0.0055 | 0.116 |
| SFRP1_2 | TTTTTTTTTTCTTTTTTTTTTTTTT | 0.0053 | 0.0049 | 0.629 |
| SFRP1_2 | TTTTTTTTTTTTTCTTTTTTTTTTT | 0.0045 | 0.0055 | 0.200 |
| SFRP1_2 | TTTTTTTTTTTTTTTTTTTTTTTTC | 0.0036 | 0.0039 | 0.547 |
| SFRP1_2 | TTTTTTTTTTTTTTTTTTTTTCCTT | 0.0031 | 0.0027 | 0.583 |
| SFRP1_2 | TTTTTTTTTTTTTTCCTTTTTTTTT | 0.0021 | 0.0026 | 0.239 |
| SFRP1_2 | TTTTTTTTTTTTTTTTTTTTCTCTT | 0.0021 | 0.0015 | 0.210 |
| SFRP1_2 | TTTTTTTTTTTTTTTTTTTCCTTTT | 0.0011 | 0.0011 | 0.946 |
| SFRP1_2 | CTCTTTTTTTTTTTTTTTTTTTTTT | 0.0013 | 0.0008 | 0.345 |
| SFRP1_2 | TTCTTTTTTTTTTTTTTTTTTTCTT | 0.0003 | 0.0006 | 0.366 |
| SFRP1_2 | TTTTTTTTTTTTTTTTTTCCCCCTT | 0.0006 | 0.0003 | 0.517 |
| SFRP1_2 | TTTTTTTTTCTTTTTCTTTTTTTTT | 0.0003 | 0.0005 | 0.528 |
| SFRP1_2 | CTTTTTTTTTTTTTTTTTTTCTTTT | 0.0003 | 0.0001 | 0.429 |
| SFRP1_2 | TTTTTTTTCTTTTTTTTTTTTCTTT | 0.0001 | 0.0023 | 0.300 |
| WNT3A_1 | TTTTTTTTTTTTTT | 0.8380 | 0.8328 | 0.216 |
| WNT3A_1 | TTTTTTTTTTTTTC | 0.0171 | 0.0192 | **0.013** |
| WNT3A_1 | TTTTTTTTTTTCTT | 0.0152 | 0.0129 | 0.158 |
| WNT3A_1 | TTTTTTCTTTTTTT | 0.0132 | 0.0128 | 0.501 |
| WNT3A_1 | TTTTCTTTTTTTTT | 0.0094 | 0.0100 | 0.231 |
| WNT3A_1 | TTTTTTTTTTCTTT | 0.0099 | 0.0098 | 0.951 |
| WNT3A_1 | TTTTTTTCTTTTTT | 0.0093 | 0.0100 | 0.415 |
| WNT3A_1 | TTTTTTTTCTTTTT | 0.0079 | 0.0090 | **0.049** |
| WNT3A_1 | TTTTTTTTTTTTCT | 0.0094 | 0.0085 | 0.370 |
| WNT3A_1 | TTCTTTTTTTTTTT | 0.0073 | 0.0078 | 0.297 |
| WNT3A_1 | TTTTTTTTTCTTTT | 0.0059 | 0.0065 | 0.116 |
| WNT3A_1 | CTTTTTTTTTTTTT | 0.0085 | 0.0066 | 0.257 |
| WNT3A_1 | TCTTTTTTTTTTTT | 0.0058 | 0.0064 | 0.154 |
| WNT3A_1 | TTTTTCTTTTTTTT | 0.0060 | 0.0061 | 0.778 |
| WNT3A_1 | TTTTTTTTTTTTCC | 0.0008 | 0.0005 | 0.470 |
| WNT3A_1 | TTTTTTCTCTTTTT | 0.0005 | 0.0009 | 0.414 |
| WNT3A_2 | TTTTTTTTTTTTTTTTTTTTT | 0.6833 | 0.6737 | 0.348 |
| WNT3A_2 | TTCTTTTTTTTTTTTTTTTTT | 0.0233 | 0.0204 | 0.180 |
| WNT3A_2 | TTTTTTTTTTTTTTTTTTTCT | 0.0139 | 0.0137 | 0.742 |
| WNT3A_2 | TTTCTTTTTTTTTTTTTTTTT | 0.0158 | 0.0152 | 0.776 |
| WNT3A_2 | TTTTTTTTTTTTCTTTTTTTT | 0.0112 | 0.015 | 0.174 |
| WNT3A_2 | TTTTTTTTTTTTTTCTTTTTT | 0.0103 | 0.0111 | 0.197 |
| WNT3A_2 | TTTTTTCTTTTTTTTTTTTTT | 0.0099 | 0.0100 | 0.937 |
| WNT3A_2 | CTTTTTTTTTTTTTTTTTTTT | 0.0097 | 0.0116 | **0.027** |
| WNT3A_2 | TTTTCTTTTTTTTTTTTTTTT | 0.0122 | 0.0109 | 0.537 |
| WNT3A_2 | TTTTTTTCTTTTTTTTTTTTT | 0.0097 | 0.0102 | 0.364 |
| WNT3A_2 | TTTTTTTTTTTTTCTTTTTTT | 0.0088 | 0.0097 | 0.126 |
| WNT3A_2 | TTTTTCTTTTTTTTTTTTTTT | 0.009 | 0.0088 | 0.639 |
| WNT3A_2 | TTTTTTTTTTTTTTTTTCTTT | 0.0088 | 0.0086 | 0.659 |
| WNT3A_2 | TTTTTTTTTTTTTTTCTTTTT | 0.0080 | 0.0087 | 0.206 |
| WNT3A_2 | TTTTTTTTTCTTTTTTTTTTT | 0.0076 | 0.0079 | 0.521 |
| WNT3A_2 | TTTTTTTTTTTTTTTTCTTTT | 0.0077 | 0.0079 | 0.714 |
| WNT3A_2 | TTTTTTTTTTCTTTTTTTTTT | 0.0072 | 0.0080 | 0.165 |
| WNT3A_2 | TTTTTTTTTTTTTTTTTTTTC | 0.0067 | 0.0067 | 0.976 |
| WNT3A_2 | TCTTTTTTTTTTTTTTTTTTT | 0.0065 | 0.0059 | 0.223 |
| WNT3A_2 | TTCTCTTTTTTTTTTTTTTTT | 0.0007 | 0.0010 | 0.220 |
| WNT3A_2 | TTTTTTTTTTTTCTTTTTTCT | 0.0003 | 0.0007 | 0.172 |
| WNT3A_2 | TTTTTTTTTTTTTTCTTCTTT | 0.0017 | 0.0004 | 0.363 |
| WNT3A_2 | TTTTTTTCCTCTTTTTTTTTT | 0.0015 | 0.0001 | 0.334 |

Bold value means *P* < 0.05.

**Table S7** The correlation between WNT signaling pathway genes methylation levels and their respective expression among PTB patients

| Clinical parameters | *SFRP1* methylation level | | *WNT3A* methylation level | | *CTNNB1* methylation level | | *DKK-1* methylation level | | *LRP6* methylation level | |
| --- | --- | --- | --- | --- | --- | --- | --- | --- | --- | --- |
|  | *r_s_* | *P* value | *r_s_* | *P* value | *r_s_* | *P* value | *r_s_* | *P* value | *r_s_* | *P* value |
| *SFRP1* expression level | 0.113 | 0.467 | - | - | - | - | - | - | - | - |
| *WNT3A* expression level | - | - | -0.086 | 0.580 | - | - | - | - | - | - |
| *CTNNB1* expression level | - | - | - | - | 0.229 | 0.134 | - | - | - | - |
| *DKK-1* expression level | - | - | - | - | - | - | -0.276 | 0.070 | - | - |
| *LRP6*  expression level | - | - | - | - | - | - | - | - | 0.099 | 0.522 |

*r_s_*：Spearman’s rank correlation coefficient
